# Supplementary material for: Influence of Extrudate-Based Textural Properties on Pellet Molding Quality
Source: Pharmaceuticals (Basel). 2023 Oct 23;16(10):1505. doi: 10.3390/ph16101505 (PMC10610123; doi:10.3390/ph16101505)
Supplement: Supplementary file 1 [file pharmaceuticals-16-01505-s001.zip › pharmaceuticals-2636072-supplementary.pdf]

Supporting Information

Influence of Extrudate-Based Textural Properties on Pellet Molding Quality

Wenxiu Tian [1,†], Xue Li [1,†], Wenjie Li [1], Aile Xue [1], Minyue Zheng [1], Xiao Lin [2,*] and Yanlong Hong [1,*]

[1] Shanghai Innovation Center of TCM Health Service, Shanghai University of Traditional Chinese Medicine, No. 1200, Cai-Lun Road, Pudong District, Shanghai 201203, China; 22021554@shutcm.edu.cn (W.T.); m15242054511@163.com (X.L.); liwenjie@shutcm.edu.cn (W.L.); 22022550@shutcm.edu.cn (A.X.); zmy2290677028@126.com (M.Z.)

[2] College of Chinese Materia Medica, Shanghai University of Traditional Chinese Medicine, No. 1200, Cai-Lun Road, Pudong District, Shanghai 201203, China

* Correspondence: linxiao@shutcm.edu.cn (X.L.); hongyanlong@shutcm.edu.cn (Y.H.)

† These authors contributed equally to this work.

Table S1. R values and classification of extrusion-spheronization of 300 prescriptions containing single TCM extracts (n=5, x ± s).

No.	Model drug	Drug loading （%）	Water added (%)	$\overline{R}$	Extrusion results	Rounding results
1	*Artemisiae Argyi Folium*	20	55	1.22±0.04	E1	S1
2	*Cynanchi Stauntonii Rhizoma Et Radix*	20	55	1.30±0.08	E1	S1
3	*Atractylodis Macrocephalae Rhizoma*	20	55	1.24±0.10	E1	S1
4	*Patriniae Herba*	20	75	1.31±0.09	E1	S1
5	*Bupleuri Radix*	20	45	1.32±0.08	E1	S1
6	*Bupleuri Radix*	20	55	1.33±0.08	E1	S1
7	*Rhei Radix Et Rhizoma*	30	40	1.22±0.04	E1	S1
8	*Zingiberis Rhizoma*	20	45	1.34±0.09	E1	S1
9	*Zingiberis Rhizoma*	20	55	1.22±0.11	E1	S1
10	*Sargentodoxae Caulis*	20	65	1.30±0.06	E1	S1
11	*Sophorae Flavescentis Radix*	30	35	1.24±0.06	E1	S1
12	*Farfarae Flos*	20	55	1.40±0.06	E1	S1
13	*Farfarae Flos*	30	35	1.21±0.03	E1	S1
14	*Eriobotryae Folium*	20	50	1.30±0.09	E1	S1
15	*Cnidii Fructus*	20	45	1.29±0.10	E1	S1
16	*Gastrodiae Rhizoma*	30	40	1.25±0.08	E1	S1
17	*Siegesbeckiae Herba*	20	65	1.22±0.05	E1	S1
18	*Magnoliae Flos*	20	55	1.23±0.06	E1	S1
19	*Magnoliae Flos*	20	65	1.21±0.05	E1	S1
20	*Leonuri Herba*	20	60	1.24±0.11	E1	S1
21	*Artemisiae Argyi Folium*	40	25	1.12±0.10	E2	S2
22	*Patriniae Herba*	30	40	1.16±0.03	E2	S2
23	*Arecae Semen*	30	40	1.17±0.03	E2	S2
24	*Citri Reticulatae Pericarpium*	20	45	1.18±0.06	E2	S2
25	*Citri Reticulatae Pericarpium*	40	25	1.15±0.04	E2	S2
26	*Citri Reticulatae Pericarpium*	40	35	1.15±0.05	E2	S2
27	*Rhei Radix Et Rhizoma*	40	30	1.16±.056	E2	S2
28	*Rhei Radix Et Rhizoma*	40	40	1.17±0.04	E2	S2
29	*Eucommiae Cortex*	30	30	1.14±0.07	E2	S2
30	*Eucommiae Cortex*	30	40	1.18±0.03	E2	S2
31	*Eucommiae Cortex*	40	35	1.18±0.04	E2	S2
32	*Eucommiae Cortex*	40	30	1.05±0.02	E2	S2
33	*Zingiberis Rhizoma*	30	45	1.18±0.02	E2	S2
34	*Sargentodoxae Caulis*	30	35	1.15±0.03	E2	S2
35	*Astragali Radix*	30	25	1.12±0.04	E2	S2
36	*Astragali Radix*	40	25	1.19±0.01	E2	S2
37	*Sophorae Flavescentis Radix*	20	55	1.11±0.02	E2	S2
38	*Sophorae Flavescentis Radix*	40	30	1.17±0.04	E2	S2
39	*Sophorae Flavescentis Radix*	40	20	1.15±0.04	E2	S2
40	*Eriobotryae Folium*	30	40	1.15±0.03	E2	S2
41	*Eriobotryae Folium*	40	30	1.15±0.02	E2	S2
42	*Cnidii Fructus*	30	35	1.13±0.10	E2	S2
43	*Cnidii Fructus*	40	20	1.17±0.06	E2	S2
44	*Acori Tatarinowii Rhizoma*	20	45	1.19±0.03	E2	S2
45	*Acori Tatarinowii Rhizoma*	30	30	1.07±0.05	E2	S2
46	*Acori Tatarinowii Rhizoma*	30	40	1.13±0.04	E2	S2
47	*Acori Tatarinowii Rhizoma*	40	25	1.16±0.03	E2	S2
48	*Gastrodiae Rhizoma*	40	25	1.19±0.01	E2	S2
49	*Siegesbeckiae Herba*	30	40	1.16±0.04	E2	S2
50	*Siegesbeckiae Herba*	40	25	1.14±0.05	E2	S2

51	*Chrysanthemi Indici Flos*	30	50	1.20±0.04	E2	S2
52	*Chrysanthemi Indici Flos*	40	30	1.10±0.05	E2	S2
53	*Chrysanthemi Indici Flos*	40	35	1.15±0.05	E2	S2
54	*Gardeniae Fructus*	20	50	1.12±0.07	E2	S2
55	*Gardeniae Fructus*	30	35	1.17±0.08	E2	S2
56	*Gardeniae Fructus*	30	45	1.17±0.07	E2	S2
57	*Gardeniae Fructus*	40	25	1.12±0.06	E2	S2
58	*Gardeniae Fructus*	40	35	1.14±0.05	E2	S2
59	*Artemisiae Argyi Folium*	20	65	1.28±0.06	E3	S3
60	*Artemisiae Argyi Folium*	20	75	1.28±0.07	E3	S3
61	*Artemisiae Argyi Folium*	20	85	1.33±0.06	E3	S3
62	*Artemisiae Argyi Folium*	30	40	1.30±0.04	E3	S3
63	*Artemisiae Argyi Folium*	30	50	1.36±0.18	E3	S3
64	*Artemisiae Argyi Folium*	30	60	1.28±0.08	E3	S3
65	*Artemisiae Argyi Folium*	40	35	1.27±0.10	E3	S3
66	*Artemisiae Argyi Folium*	40	45	1.32±0.08	E3	S3
67	*Cynanchi Stauntonii Rhizoma Et Radix*	20	65	1.32±0.11	E3	S3
68	*Cynanchi Stauntonii Rhizoma Et Radix*	20	75	1.33±0.11	E3	S3
69	*Cynanchi Stauntonii Rhizoma Et Radix*	30	40	1.32±0.07	E3	S3
70	*Cynanchi Stauntonii Rhizoma Et Radix*	30	50	1.34±0.04	E3	S3
71	*Cynanchi Stauntonii Rhizoma Et Radix*	30	60	1.38±0.07	E3	S3
72	*Cynanchi Stauntonii Rhizoma Et Radix*	40	40	1.31±0.09	E3	S3
73	*Cynanchi Stauntonii Rhizoma Et Radix*	40	45	1.28±0.07	E3	S3
74	*Atractylodis Macrocephalae Rhizoma*	20	65	1.33±0.10	E3	S3
75	*Atractylodis Macrocephalae Rhizoma*	20	75	1.25±0.03	E3	S3
76	*Atractylodis Macrocephalae Rhizoma*	20	85	1.32±0.11	E3	S4
77	*Atractylodis Macrocephalae Rhizoma*	30	55	1.33±0.10	E3	S3
78	*Atractylodis Macrocephalae Rhizoma*	30	65	1.28±0.04	E3	S4
79	*Atractylodis Macrocephalae Rhizoma*	40	35	1.35±0.07	E3	S3
80	*Atractylodis Macrocephalae Rhizoma*	40	40	1.35±0.07	E3	S3
81	*Patriniae Herba*	20	85	1.27±0.07	E3	S3
82	*Patriniae Herba*	20	95	1.27±0.04	E3	S3
83	*Patriniae Herba*	30	50	1.28±0.11	E3	S3
84	*Patriniae Herba*	30	60	1.32±0.09	E3	S3
85	*Patriniae Herba*	40	45	1.32±0.07	E3	S3
86	*Patriniae Herba*	40	50	1.35±0.12	E3	S3
87	*Patriniae Herba*	40	55	1.33±0.07	E3	S3
88	*Arecae Semen*	20	65	1.26±0.06	E3	S3
89	*Arecae Semen*	20	75	1.28±0.04	E3	S3
90	*Arecae Semen*	20	85	1.40±0.09	E3	S3
91	*Arecae Semen*	30	50	1.28±0.04	E3	S3
92	*Arecae Semen*	30	60	1.32±0.04	E3	S3
93	*Arecae Semen*	40	35	1.29±0.10	E3	S3
94	*Arecae Semen*	40	45	1.40±0.09	E3	S4
95	*Bupleuri Radix*	20	65	1.49±0.10	E3	S3
96	*Bupleuri Radix*	20	75	1.25±0.07	E3	S3
97	*Bupleuri Radix*	30	40	1.24±0.03	E3	S3
98	*Bupleuri Radix*	30	50	1.31±0.06	E3	S3
99	*Bupleuri Radix*	30	60	1.26±0.12	E3	S3
100	*Bupleuri Radix*	30	70	1.27±0.07	E3	S4
101	*Bupleuri Radix*	40	30	1.29±0.13	E3	S3
102	*Bupleuri Radix*	40	35	1.34±0.08	E3	S3
103	*Bupleuri Radix*	40	45	1.33±0.10	E3	S4
104	*Citri Reticulatae Pericarpium*	20	55	1.26±0.05	E3	S3

105	*Citri Reticulatae Pericarpium*	20	65	1.30±0.06	E3	S3
106	*Citri Reticulatae Pericarpium*	20	75	1.36±0.06	E3	S3
107	*Citri Reticulatae Pericarpium*	30	45	1.30±0.04	E3	S3
108	*Citri Reticulatae Pericarpium*	30	55	1.33±0.05	E3	S3
109	*Citri Reticulatae Pericarpium*	30	65	1.29±0.06	E3	S4
110	*Citri Reticulatae Pericarpium*	40	45	1.31±0.06	E3	S3
111	*Rhei Radix Et Rhizoma*	20	65	1.28±0.04	E3	S3
112	*Rhei Radix Et Rhizoma*	20	75	1.27±0.05	E3	S3
113	*Rhei Radix Et Rhizoma*	20	85	1.31±0.09	E3	S3
114	*Rhei Radix Et Rhizoma*	30	50	1.34±0.09	E3	S3
115	*Rhei Radix Et Rhizoma*	30	60	1.24±0.04	E3	S3
116	*Rhei Radix Et Rhizoma*	30	70	1.28±0.06	E3	S3
117	*Rhei Radix Et Rhizoma*	40	50	1.25±0.02	E3	S3
118	*Eucommiae Cortex*	20	70	1.25±0.06	E3	S3
119	*Eucommiae Cortex*	20	80	1.25±0.01	E3	S3
120	*Eucommiae Cortex*	20	85	1.31±0.12	E3	S3
121	*Eucommiae Cortex*	20	90	1.24±0.03	E3	S4
122	*Eucommiae Cortex*	30	50	1.26±0.10	E3	S3
123	*Eucommiae Cortex*	30	60	1.26±0.12	E3	S3
124	*Eucommiae Cortex*	40	40	1.29±0.15	E3	S3
125	*Zingiberis Rhizoma*	20	65	1.35±0.09	E3	S3
126	*Zingiberis Rhizoma*	30	55	1.46±0.07	E3	S3
127	*Zingiberis Rhizoma*	30	65	1.31±0.11	E3	S3
128	*Zingiberis Rhizoma*	40	35	1.30±0.05	E3	S2
129	*Zingiberis Rhizoma*	40	45	1.36±0.03	E3	S3
130	*Sargentodoxae Caulis*	20	75	1.38±0.09	E3	S3
131	*Sargentodoxae Caulis*	20	85	1.36±0.01	E3	S3
132	*Sargentodoxae Caulis*	30	45	1.31±0.14	E3	S3
133	*Sargentodoxae Caulis*	30	55	1.31±0.06	E3	S3
134	*Sargentodoxae Caulis*	30	65	1.39±0.09	E3	S3
135	*Sargentodoxae Caulis*	40	40	1.29±0.05	E3	S3
136	*Sargentodoxae Caulis*	40	50	1.31±0.09	E3	S3
137	*Astragali Radix*	20	60	1.28±0.11	E3	S3
138	*Astragali Radix*	20	70	1.34±0.12	E3	S3
139	*Astragali Radix*	20	80	1.32±0.09	E3	S3
140	*Astragali Radix*	20	90	1.32±0.06	E3	S4
141	*Astragali Radix*	30	35	1.35±0.12	E3	S3
142	*Astragali Radix*	30	45	1.27±0.04	E3	S3
143	*Astragali Radix*	40	30	1.27±0.11	E3	S2
144	*Astragali Radix*	40	40	1.40±0.04	E3	S3
145	*Astragali Radix*	40	45	1.32±0.06	E3	S4
146	*Scutellariae Radix*	20	60	1.32±0.10	E3	S3
147	*Scutellariae Radix*	20	70	1.37±0.08	E3	S3
148	*Scutellariae Radix*	20	75	1.25±0.09	E3	S3
149	*Scutellariae Radix*	20	80	1.33±0.09	E3	S3
150	*Scutellariae Radix*	30	35	1.35±0.12	E3	S2
151	*Scutellariae Radix*	30	45	1.24±0.07	E3	S3
152	*Scutellariae Radix*	30	50	1.26±0.04	E3	S3
153	*Scutellariae Radix*	30	55	1.24±0.02	E3	S3
154	*Scutellariae Radix*	40	40	1.26±0.03	E3	S3
155	*Sophorae Flavescentis Radix*	20	65	1.30±0.09	E3	S3
156	*Sophorae Flavescentis Radix*	20	75	1.27±0.09	E3	S3
157	*Sophorae Flavescentis Radix*	30	45	1.30±0.08	E3	S3
158	*Sophorae Flavescentis Radix*	30	55	1.38±0.11	E3	S3

159	*Sophorae Flavescentis Radix*	40	40	1.27±0.01	E3	S3
160	*Sophorae Flavescentis Radix*	40	50	1.33±0.06	E3	S3
161	*Farfarae Flos*	20	65	1.35±0.09	E3	S3
162	*Farfarae Flos*	20	75	1.30±0.07	E3	S3
163	*Farfarae Flos*	20	85	1.27±0.07	E3	S3
164	*Farfarae Flos*	30	45	1.32±0.06	E3	S3
165	*Farfarae Flos*	30	55	1.31±0.11	E3	S3
166	*Farfarae Flos*	30	65	1.27±0.05	E3	S3
167	*Farfarae Flos*	40	35	1.23±0.07	E3	S2
168	*Farfarae Flos*	40	40	1.32±0.06	E3	S3
169	*Farfarae Flos*	40	45	1.36±0.11	E3	S3
170	*Eriobotryae Folium*	20	60	1.26±0.07	E3	S3
171	*Eriobotryae Folium*	20	70	1.26±0.07	E3	S3
172	*Eriobotryae Folium*	20	80	1.26±0.04	E3	S3
173	*Eriobotryae Folium*	30	50	1.28±0.03	E3	S3
174	*Eriobotryae Folium*	30	60	1.33±0.11	E3	S3
175	*Eriobotryae Folium*	40	40	1.28±0.09	E3	S2
176	*Eriobotryae Folium*	40	45	1.31±0.11	E3	S4
177	*Cnidii Fructus*	20	55	1.38±0.11	E3	S3
178	*Cnidii Fructus*	20	65	1.25±0.03	E3	S3
179	*Cnidii Fructus*	20	75	1.26±0.05	E3	S3
180	*Cnidii Fructus*	30	45	1.26±0.02	E3	S3
181	*Cnidii Fructus*	30	55	1.27±0.05	E3	S3
182	*Cnidii Fructus*	40	30	1.34±0.12	E3	S3
183	*Cnidii Fructus*	40	40	1.30±0.12	E3	S3
184	*Acori Tatarinowii Rhizoma*	20	55	1.31±0.03	E3	S2
185	*Acori Tatarinowii Rhizoma*	20	65	1.25±0.03	E3	S3
186	*Acori Tatarinowii Rhizoma*	20	75	1.26±0.04	E3	S3
187	*Acori Tatarinowii Rhizoma*	30	50	1.31±0.09	E3	S3
188	*Acori Tatarinowii Rhizoma*	40	35	1.30±0.04	E3	S3
189	*Gastrodiae Rhizoma*	20	60	1.28±0.07	E3	S3
190	*Gastrodiae Rhizoma*	20	70	1.29±0.07	E3	S3
191	*Gastrodiae Rhizoma*	20	80	1.34±0.13	E3	S3
192	*Gastrodiae Rhizoma*	20	90	1.32±0.02	E3	S3
193	*Gastrodiae Rhizoma*	30	50	1.27±0.05	E3	S3
194	*Gastrodiae Rhizoma*	30	60	1.26±0.06	E3	S3
195	*Gastrodiae Rhizoma*	30	70	1.31±0.06	E3	S4
196	*Gastrodiae Rhizoma*	40	35	1.28±0.11	E3	S3
197	*Gastrodiae Rhizoma*	40	45	1.26±0.03	E3	S3
198	*Schisandrae Chinensis Fructus*	20	65	1.27±0.04	E3	S3
199	*Schisandrae Chinensis Fructus*	20	75	1.26±0.03	E3	S3
200	*Schisandrae Chinensis Fructus*	20	80	1.28±0.06	E3	S3
201	*Schisandrae Chinensis Fructus*	20	85	1.24±0.01	E3	S3
202	*Schisandrae Chinensis Fructus*	30	45	1.29±0.11	E3	S3
203	*Schisandrae Chinensis Fructus*	30	55	1.27±0.06	E3	S3
204	*Schisandrae Chinensis Fructus*	30	60	1.28±0.11	E3	S3
205	*Schisandrae Chinensis Fructus*	40	30	1.25±0.06	E3	S2
206	*Schisandrae Chinensis Fructus*	40	35	1.27±0.03	E3	S3
207	*Schisandrae Chinensis Fructus*	40	40	1.32±0.07	E3	S4
208	*Schisandrae Chinensis Fructus*	40	45	1.29±0.10	E3	S3
209	*Siegesbeckiae Herba*	20	75	1.26±0.03	E3	S3
210	*Siegesbeckiae Herba*	20	85	1.29±0.08	E3	S3
211	*Siegesbeckiae Herba*	20	95	1.29±0.08	E3	S3
212	*Siegesbeckiae Herba*	30	50	1.27±0.05	E3	S3

213	*Siegesbeckiae Herba*	30	60	1.28±0.05	E3	S3
214	*Siegesbeckiae Herba*	40	35	1.26±0.05	E3	S2
215	*Siegesbeckiae Herba*	40	45	1.34±0.11	E3	S3
216	*Magnoliae Flos*	20	75	1.33±0.09	E3	S3
217	*Magnoliae Flos*	20	85	1.30±0.06	E3	S3
218	*Magnoliae Flos*	30	60	1.27±0.08	E3	S3
219	*Magnoliae Flos*	30	65	1.31±0.10	E3	S3
220	*Magnoliae Flos*	30	70	1.27±0.07	E3	S3
221	*Magnoliae Flos*	40	45	1.28±0.11	E3	S3
222	*Magnoliae Flos*	40	50	1.28±0.07	E3	S4
223	*Chrysanthemi Indici Flos*	20	60	1.28±0.09	E3	S3
224	*Chrysanthemi Indici Flos*	20	70	1.30±0.09	E3	S3
225	*Chrysanthemi Indici Flos*	20	80	1.26±0.09	E3	S3
226	*Chrysanthemi Indici Flos*	20	90	1.26±0.05	E3	S4
227	*Chrysanthemi Indici Flos*	30	60	1.29±0.12	E3	S3
228	*Chrysanthemi Indici Flos*	30	70	1.29±0.04	E3	S3
229	*Chrysanthemi Indici Flos*	40	45	1.24±0.05	E3	S3
230	*Leonuri Herba*	20	70	1.31±0.01	E3	S3
231	*Leonuri Herba*	20	80	1.31±0.12	E3	S3
232	*Leonuri Herba*	20	90	1.33±0.15	E3	S3
233	*Leonuri Herba*	30	45	1.35±0.02	E3	S3
234	*Leonuri Herba*	30	55	1.28±0.15	E3	S3
235	*Leonuri Herba*	30	65	1.33±0.06	E3	S3
236	*Leonuri Herba*	40	30	1.35±0.10	E3	S2
237	*Leonuri Herba*	40	40	1.42±0.11	E3	S3
238	*Gardeniae Fructus*	20	65	1.24±0.04	E3	S3
239	*Gardeniae Fructus*	20	60	1.26±0.04	E3	S3
240	*Gardeniae Fructus*	20	70	1.27±0.07	E3	S3
241	*Gardeniae Fructus*	30	50	1.25±0.06	E3	S3
242	*Gardeniae Fructus*	30	55	1.26±0.09	E3	S3
243	*Artemisiae Argyi Folium*	30	70	1.20±0.11	E4	S4
244	*Artemisiae Argyi Folium*	40	55	1.17±0.12	E4	S4
245	*Cynanchi Stauntonii Rhizoma Et Radix*	20	85	1.12±0.05	E4	S4
246	*Cynanchi Stauntonii Rhizoma Et Radix*	30	70	1.16±0.04	E4	S4
247	*Cynanchi Stauntonii Rhizoma Et Radix*	40	50	1.13±0.06	E4	S4
248	*Cynanchi Stauntonii Rhizoma Et Radix*	40	60	1.09±0.05	E4	S4
249	*Atractylodis Macrocephalae Rhizoma*	30	85	1.15±0.07	E4	S4
250	*Atractylodis Macrocephalae Rhizoma*	30	75	1.19±0.02	E4	S4
251	*Atractylodis Macrocephalae Rhizoma*	40	45	1.19±0.03	E4	S4
252	*Atractylodis Macrocephalae Rhizoma*	40	55	1.18±0.04	E4	S4
253	*Patriniae Herba*	20	100	1.12±0.07	E4	S4
254	*Patriniae Herba*	30	70	1.18±0.03	E4	S4
255	*Patriniae Herba*	40	60	1.14±0.02	E4	S4
256	*Arecae Semen*	20	95	1.10±0.03	E4	S4
257	*Arecae Semen*	30	70	1.18±0.03	E4	S4
258	*Arecae Semen*	40	55	1.14±0.02	E4	S4
259	*Arecae Semen*	40	60	1.13±0.06	E4	S4
260	*Bupleuri Radix*	40	55	1.18±0.03	E4	S4
261	*Citri Reticulatae Pericarpium*	30	75	1.18±0.03	E4	S4
262	*Citri Reticulatae Pericarpium*	40	55	1.11±0.03	E4	S4
263	*Rhei Radix Et Rhizoma*	20	95	1.17±0.02	E4	S4
264	*Rhei Radix Et Rhizoma*	40	60	1.13±0.06	E4	S4
265	*Eucommiae Cortex*	40	50	1.13±0.05	E4	S4
266	*Zingiberis Rhizoma*	20	75	1.19±0.02	E4	S4

267	*Zingiberis Rhizoma*	30	75	1.17±0.06	E4	S4
268	*Zingiberis Rhizoma*	40	55	1.16±0.07	E4	S4
269	*Zingiberis Rhizoma*	40	65	1.12±0.08	E4	S4
270	*Sargentodoxae Caulis*	20	95	1.08±0.06	E4	S4
271	*Sargentodoxae Caulis*	40	65	1.12±0.03	E4	S4
272	*Sargentodoxae Caulis*	40	60	1.17±0.06	E4	S4
273	*Astragali Radix*	30	55	1.17±0.07	E4	S4
274	*Scutellariae Radix*	40	45	1.16±0.04	E4	S4
275	*Scutellariae Radix*	40	50	1.12±0.04	E4	S4
276	*Scutellariae Radix*	40	55	1.18±0.01	E4	S4
277	*Sophorae Flavescentis Radix*	20	85	1.16±0.04	E4	S4
278	*Sophorae Flavescentis Radix*	30	65	1.19±0.03	E4	S4
279	*Farfarae Flos*	40	55	1.16±0.07	E4	S4
280	*Eriobotryae Folium*	30	70	1.15±0.04	E4	S4
281	*Eriobotryae Folium*	40	50	1.18±0.09	E4	S4
282	*Cnidii Fructus*	30	65	1.18±0.05	E4	S4
283	*Cnidii Fructus*	40	50	1.20±0.09	E4	S4
284	*Acori Tatarinowii Rhizoma*	30	60	1.19±0.01	E4	S4
285	*Acori Tatarinowii Rhizoma*	40	45	1.19±0.05	E4	S4
286	*Acori Tatarinowii Rhizoma*	40	55	1.12±0.04	E4	S4
287	*Gastrodiae Rhizoma*	40	55	1.17±0.09	E4	S4
288	*Schisandrae Chinensis Fructus*	30	65	1.17±0.04	E4	S4
289	*Siegesbeckiae Herba*	30	70	1.13±0.08	E4	S4
290	*Siegesbeckiae Herba*	40	55	1.19±0.02	E4	S4
291	*Magnoliae Flos*	30	75	1.19±0.05	E4	S4
292	*Magnoliae Flos*	40	55	1.17±0.07	E4	S4
293	*Magnoliae Flos*	40	65	1.13±0.06	E4	S4
294	*Chrysanthemi Indici Flos*	30	80	1.16±0.03	E4	S4
295	*Chrysanthemi Indici Flos*	40	40	1.14±0.01	E4	S4
296	*Leonuri Herba*	30	75	1.19±0.06	E4	S4
297	*Leonuri Herba*	40	50	1.16±0.03	E4	S4
298	*Leonuri Herba*	40	60	1.15±0.02	E4	S4
299	*Gardeniae Fructus*	40	50	1.16±0.02	E4	S4
300	*Gardeniae Fructus*	40	45	1.18±0.03	E4	S4

Notes—R: Surface roughness of the extrudates. E1: Extrudate cannot be extruded smoothly and the total amount of extrudate obtained is less than half of the amount fed. E2: Extrudate can be extruded smoothly with smooth surface. E3: Extrudate can be extruded smoothly with rough surface. E4: Extrudate can be extruded smoothly. But it's clumped together. S1: The extrudate after rounding becomes fine powder. S2: The extrudate is rounded to obtain pellets in the form of rods, dumbbells or double balls. S3: The extrudate is rounded to obtain pellets in the form of spherical. S4: The extrudate after rounding becomes the large ball or irregularly shaped mass.

Table S2. Experimental results for the determination of the physical properties of the extrudates with single TCM prescription (*n*=3, x ± s).

No.	Model drug	Drug loading (%)	Water added (%)	Ha(g)	Ad (g.s)	Sp	Co	Ch	Re
1	*Cynanchi Stauntonii Rhizoma Et Radix*	20	55	19,343.41±1049.31	-5.58±2.85	0.42±0.02	0.39±0.02	3122.80±295.63	0.12±0.00
2	*Cynanchi Stauntonii Rhizoma Et Radix*	20	65	18,125.04±261.10	-23.93±14.09	0.38±0.00	0.37±0.01	2541.14±112.38	0.12±0.00
3	*Cynanchi Stauntonii Rhizoma Et Radix*	20	75	16,974.54±1028.16	-12.91±2.56	0.43±0.01	0.36±0.01	2622.23±174.48	0.12±0.00
4	*Cynanchi Stauntonii Rhizoma Et Radix*	20	85	12,928.35±426.61	-51.76±9.36	0.99±0.01	0.28±0.01	3639.09±167.98	0.11±0.00
5	*Cynanchi Stauntonii Rhizoma Et Radix*	30	40	22,629.77±565.81	-10.85±2.47	0.42±0.01	0.39±0.01	3715.06±207.98	0.13±0.01
6	*Cynanchi Stauntonii Rhizoma Et Radix*	30	50	19,837.79±666.84	-9.21±1.94	0.42±0.01	0.39±0.01	3268.89±226.76	0.12±0.00
7	*Cynanchi Stauntonii Rhizoma Et Radix*	30	60	16,518.60±785.91	-18.04±1.65	0.48±0.08	0.35±0.02	2811.92±488.45	0.11±0.00
8	*Cynanchi Stauntonii Rhizoma Et Radix*	30	70	13,273.80±85.63	-37.48±9.34	0.58±0.13	0.31±0.00	2394.92±543.11	0.11±0.01
9	*Cynanchi Stauntonii Rhizoma Et Radix*	40	40	17,191.22±986.93	-69.21±15.82	0.47±0.03	0.35±0.01	2775.29±281.92	0.11±0.00
10	*Cynanchi Stauntonii Rhizoma Et Radix*	40	45	16,458.39±429.92	-108.02±31.11	0.68±0.27	0.34±0.00	3792.48±1551.85	0.10±0.00
11	*Cynanchi Stauntonii Rhizoma Et Radix*	40	50	12,770.77±191.07	-73.78±18.84	0.94±0.10	0.28±0.00	3298.21±275.76	0.09±0.00
12	*Cynanchi Stauntonii Rhizoma Et Radix**	40	60	19,443.08±1390.02	-321.89±42.53	0.83±0.14	0.40±0.02	6435.88±1202.06	0.23±0.01
13	*Sargentodoxae Caulis*	20	65	19,263.06±1756.23	-7.77±2.62	0.39±0.02	0.36±0.01	2747.51±141.18	0.11±0.00
14	*Sargentodoxae Caulis*	20	75	17,722.79±1352.22	-11.84±1.47	0.41±0.02	0.35±0.01	2564.13±387.90	0.11±0.00
15	*Sargentodoxae Caulis*	20	85	16,193.19±1431.11	-13.26±0.62	0.44±0.04	0.35±0.00	2460.36±445.42	0.11±0.00
16	*Sargentodoxae Caulis*	20	95	12,442.61±220.74	-42.79±6.29	0.79±0.04	0.28±0.01	2416.44±150.04	0.09±0.00
17	*Sargentodoxae Caulis*	30	35	22,136.13±271.45	-16.71±4.48	0.41±0.00	0.48±0.01	3472.74±168.88	0.11±0.00
18	*Sargentodoxae Caulis*	30	45	16,550.42±584.42	-13.26±61.63	0.46±0.00	0.34±0.01	2573.97±165.18	0.11±0.00
19	*Sargentodoxae Caulis*	30	55	19,463.89±961.40	-14.84±0.32	0.41±0.12	0.36±0.01	2898.31±53.23	0.10±0.01
20	*Sargentodoxae Caulis*	30	65	16,586.51±81.70	-19.80±39.94	0.47±0.00	0.34±0.01	2666.38±99.56	0.10±0.00
21	*Sargentodoxae Caulis*	40	40	22,133.60±674.71	-35.46±4.16	0.43±0.02	0.36±0.01	3394.75±251.88	0.10±0.00
22	*Sargentodoxae Caulis*	40	50	15,819.03±536.77	-42.35±4.34	0.48±0.03	0.33±0.00	2482.95±144.53	0.09±0.00
23	*Sargentodoxae Caulis*	40	60	12,100.48±316.50	-67.98±8.21	0.86±0.06	0.27±0.01	2760.49±372.44	0.08±0.00
24	*Sargentodoxae Caulis*	40	65	12,014.70±755.03	-115.80±2.11	0.98±0.01	0.23±0.01	2725.52±107.98	0.08±0.00
25	*Arecae Semen*	20	65	18,636.82±240.63	-8.50±7.42	0.54±0.13	0.33±0.01	3318.40±769.36	0.12±0.01
26	*Arecae Semen*	20	75	17,935.28±350.96	-10.30±0.97	0.45±0.04	0.36±0.01	2891.09±111.08	0.11±0.00
27	*Arecae Semen*	20	85	16,237.32±1270.80	-15.35±3.70	0.53±0.12	0.34±0.02	2900.91±578.05	0.11±0.00
28	*Arecae Semen*	20	95	13,639.58±618.96	-34.62±3.75	0.78±0.11	0.29±0.01	3088.82±302.77	0.09±0.00
29	*Arecae Semen*	30	40	21,157.59±71.89	-22.00±9.71	0.44±0.02	0.39±0.02	3673.66±314.37	0.13±0.00
30	*Arecae Semen*	30	50	18,528.81±1027.42	-21.69±8.86	0.44±0.02	0.35±0.01	2874.93±267.97	0.11±0.00
31	*Arecae Semen*	30	60	15,059.54±125.18	-31.38±8.73	0.65±0.10	0.34±0.02	3331.89±350.90	0.11±0.00
32	*Arecae Semen*	30	70	12,644.52±71.81	-7.34±0.67	0.88±0.02	0.27±0.02	3001.54±314.60	0.10±0.00
33	*Arecae Semen*	40	35	20,281.05±2188.52	-47.06±69.72	0.46±0.00	0.38±0.03	3848.24±1951.31	0.11±0.03
34	*Arecae Semen*	40	45	16,211.25±141.05	-51.56±14.00	0.60±0.05	0.35±0.01	3854.06±77.47	0.11±0.00
35	*Arecae Semen*	40	55	13,348.82±523.43	-31.35±7.91	0.52±0.04	0.32±0.01	2194.02±55.65	0.10±0.00
36	*Arecae Semen*	40	60	11,802.37±343.87	-54.98±12.09	0.78±0.08	0.27±0.01	2482.09±353.78	0.10±0.00
37	*Zingiberis Rhizoma*	20	45	24,609.78±1070.92	-13.15±1.98	0.38±0.03	0.36±0.00	3359.50±120.58	0.12±0.00
38	*Zingiberis Rhizoma*	20	55	21,405.26±722.03	-15.93±2.80	0.44±0.03	0.37±0.02	3463.47±403.95	0.12±0.00
39	*Zingiberis Rhizoma*	20	65	14,411.52±973.29	-19.31±1.65	0.52±0.04	0.34±0.03	2542.88±204.76	0.11±0.00
40	*Zingiberis Rhizoma*	20	75	13,051.56±1263.59	-30.00±3.98	0.71±0.07	0.31±0.03	2818.71±300.61	0.10±0.00

41	*Zingiberis Rhizoma*	30	45	21,129.41±1300.36	-45.84±6.42	0.57±0.13	0.35±0.01	4124.71±624.60	0.11±0.01
42	*Zingiberis Rhizoma*	30	55	14,608.99±1539.94	-118.72±41.26	0.83±0.27	0.32±0.02	3687.05±939.15	0.09±0.01
43	*Zingiberis Rhizoma*	30	65	11,894.10±820.39	-137.13±42.42	1.00±0.00	0.27±0.02	3222.80±419.42	0.09±0.00
44	*Zingiberis Rhizoma**	30	75	14,351.20±326.43	-401.95±27.23	0.94±0.09	0.29±0.01	3883.80±287.86	0.12±0.00
45	*Zingiberis Rhizoma*	40	35	10,968.32±605.41	-208.65±37.73	0.78±0.31	0.29±0.01	2408.43±888.12	0.07±0.01
46	*Zingiberis Rhizoma*	40	45	10,038.71±191.67	-412.08±73.80	0.98±0.03	0.28±0.01	2735.22±112.42	0.08±0.01
47	*Zingiberis Rhizoma*	40	55	10,029.12±515.86	-403.18±60.13	0.99±0.01	0.28±0.02	2813.03±318.78	0.09±0.01
48	*Zingiberis Rhizoma**	40	65	99,69.04±310.62	-370.13±22.63	0.99±0.00	0.26±0.01	2538.47±156.07	0.08±0.01
49	*Acori Tatarinowii Rhizoma*	20	45	23,044.10±675.33	-6.94±2.62	0.38±0.00	0.37±0.02	3267.14±302.96	0.12±0.00
50	*Acori Tatarinowii Rhizoma*	20	55	20,765.30±1433.07	-10.41±3.45	0.37±0.04	0.35±0.01	2736.22±247.20	0.12±0.00
51	*Acori Tatarinowii Rhizoma*	20	65	18,239.59±1291.91	-12.02±0.99	0.50±0.07	0.36±0.02	3264.39±22.76	0.12±0.01
52	*Acori Tatarinowii Rhizoma*	20	75	16,196.08±1027.80	-21.63±0.47	0.48±0.05	0.34±0.01	2639.99±402.54	0.11±0.00
53	*Acori Tatarinowii Rhizoma*	30	30	22,321.59±165.64	-19.09±3.97	0.42±0.03	0.35±0.01	3316.44±236.87	0.11±0.00
54	*Acori Tatarinowii Rhizoma*	30	40	21,638.60±3336.30	-17.78±6.72	0.41±0.04	0.37±0.03	3303.06±981.64	0.12±0.01
55	*Acori Tatarinowii Rhizoma*	30	50	18,382.88±1808.68	-20.05±6.04	0.45±0.06	0.35±0.01	2909.99±532.97	0.11±0.00
56	*Acori Tatarinowii Rhizoma*	30	60	14,404.00±1406.29	-42.95±85.98	0.59±0.00	0.32±0.01	2857.31±994.34	0.10±0.02
57	*Acori Tatarinowii Rhizoma**	40	25	17,380.50±1000.68	-352.51±82.50	1.00±0.00	0.25±0.01	4262.48±328.64	0.08±0.00
58	*Acori Tatarinowii Rhizoma*	40	35	15,597.63±1135.89	-120.21±4.21	0.94±0.07	0.29±0.00	4240.19±547.37	0.08±0.00
59	*Acori Tatarinowii Rhizoma*	40	45	12,708.72±322.69	-110.87±18.49	1.00±0.00	0.27±0.01	3459.18±66.17	0.08±0.00
60	*Acori Tatarinowii Rhizoma**	40	55	16,057.46±1503.11	-514.40±107.07	0.99±0.01	0.35±0.03	5607.72±994.57	0.18±0.03
61	*Cnidii Fructus*	20	45	27,755.38±871.54	-7.26±43.68	0.48±0.00	0.37±0.01	4990.43±227.59	0.15±0.00
62	*Cnidii Fructus*	20	55	22,223.44±770.95	-4.93±127.72	0.40±0.00	0.38±0.02	3399.13±933.35	0.13±0.02
63	*Cnidii Fructus*	20	65	17,499.99±1842.65	-6.29±3.22	0.44±0.05	0.40±0.01	3340.63±779.51	0.13±0.01
64	*Cnidii Fructus*	20	75	17,522.61±778.40	-9.95±2.16	0.43±0.05	0.39±0.01	2932.39±438.12	0.12±0.01
65	*Cnidii Fructus*	30	35	24,324.35±4156.44	-5.72±1.30	0.40±0.05	0.39±0.02	3788.10±1125.55	0.13±0.01
66	*Cnidii Fructus*	30	45	20,206.54±138.95	-6.48±2.76	0.42±0.07	0.39±0.00	3235.94±231.13	0.13±0.00
67	*Cnidii Fructus*	30	55	17,230.59±577.92	-9.15±1.89	0.44±0.01	0.37±0.02	2832.12±139.30	0.12±0.00
68	*Cnidii Fructus*	30	65	13,377.90±813.96	-75.01±10.48	0.81±0.02	0.29±0.01	3143.40±338.36	0.12±0.02
69	*Cnidii Fructus*	40	20	21,334.63±535.01	-279.58±40.87	0.50±0.09	0.33±0.02	3512.86±862.00	0.09±0.00
70	*Cnidii Fructus*	40	30	19,682.85±1242.14	-55.14±5.83	0.45±0.02	0.37±0.01	3237.13±342.22	0.11±0.00
71	*Cnidii Fructus*	40	40	14,496.09±470.75	-36.85±7.64	0.49±0.00	0.34±0.01	2443.41±110.82	0.10±0.01
72	*Cnidii Fructus*	40	50	12,101.31±533.56	-47.27±17.03	0.80±0.18	0.28±0.01	2775.65±792.39	0.10±0.00
73	*Patriniae Herba*	20	75	21,010.97±777.61	-8.91±1.01	0.42±0.02	0.39±0.00	3422.07±51.89	0.13±0.00
74	*Patriniae Herba*	20	85	17,737.00±818.21	-14.70±4.01	0.51±0.05	0.37±0.01	3309.66±178.76	0.12±0.00
75	*Patriniae Herba*	20	95	16,920.48±392.28	-19.53±3.04	0.56±0.06	0.36±0.01	3374.36±220.33	0.12±0.00
76	*Patriniae Herba*	20	100	14,601.43±511.61	-30.73±6.98	0.92±0.06	0.31±0.01	4199.92±67.33	0.11±0.00
77	*Patriniae Herba*	30	40	26,038.56±1485.50	-8.59±4.97	0.41±0.03	0.42±0.00	4481.16±155.45	0.14±0.00
78	*Patriniae Herba*	30	50	19,245.46±567.39	-10.87±1.17	0.44±0.02	0.38±0.01	3250.49±153.81	0.13±0.00
79	*Patriniae Herba*	30	60	16,807.11±501.73	-13.29±3.56	0.47±0.04	0.36±0.01	2850.37±144.02	0.12±0.01
80	*Patriniae Herba*	30	70	14,007.95±313.55	-20.48±2.46	0.63±0.07	0.32±0.00	2792.78±282.30	0.11±0.00
81	*Patriniae Herba*	40	45	21,310.39±824.43	-15.31±2.76	0.49±0.01	0.39±0.01	4043.05±198.03	0.12±0.00
82	*Patriniae Herba*	40	50	18,942.51±851.85	-15.29±2.70	0.43±0.02	0.37±0.00	2992.32±146.25	0.11±0.00
83	*Patriniae Herba*	40	55	15,980.54±396.11	-18.48±3.33	0.45±0.03	0.34±0.00	2446.38±126.60	0.11±0.00
84	*Patriniae Herba*	40	60	13,943.61±255.28	-33.78±4.24	0.84±0.07	0.30±0.01	3506.45±301.46	0.10±0.00
85	*Scutellariae Radix*	20	60	20,559.32±1132.40	-6.77±2.89	0.41±0.03	0.38±0.02	3212.04±91.65	0.12±0.01
86	*Scutellariae Radix*	20	70	18,783.25±591.95	-8.84±2.20	0.42±0.04	0.37±0.02	2943.76±542.50	0.12±0.01
87	*Scutellariae Radix*	20	75	17,645.20±432.98	-17.57±2.56	0.42±0.01	0.36±0.01	2642.44±145.26	0.12±0.00
88	*Scutellariae Radix*	20	80	16,586.14±232.10	-16.51±3.34	0.47±0.05	0.35±0.01	2740.26±211.22	0.11±0.00
89	*Scutellariae Radix*	30	35	24,027.57±327.24	-14.46±5.41	0.41±0.02	0.37±0.01	3590.28±176.98	0.11±0.01

90	*Scutellariae Radix*	30	45	19,876.29±2070.18	-17.04±2.17	0.46±0.05	0.37±0.02	3382.98±413.74	0.11±0.00
91	*Scutellariae Radix*	30	50	17,684.10±391.28	-27.42±3.17	0.43±0.02	0.36±0.02	2686.16±172.58	0.11±0.00
92	*Scutellariae Radix*	30	55	16,973.15±614.97	-19.29±3.66	0.53±0.09	0.35±0.02	3118.36±302.65	0.11±0.01
93	*Scutellariae Radix*	40	40	15,513.12±377.62	-113.10±32.09	0.90±0.17	0.31±0.01	4312.06±750.21	0.09±0.00
94	*Scutellariae Radix*	40	45	13,910.17±158.15	-56.61±2.06	0.64±0.06	0.30±0.00	2710.66±237.18	0.09±0.00
95	*Scutellariae Radix*	40	50	12,423.89±510.21	-73.70±7.71	0.98±0.01	0.28±0.01	3375.36±200.75	0.09±0.00
96	*Scutellariae Radix*	40	55	11,652.53±462.27	-84.65±13.46	1.00±0.00	0.25±0.00	2916.13±104.59	0.09±0.00
97	*Chrysanthemi Indici Flos*	20	60	21,692.30±598.13	-9.35±1.79	0.41±0.06	0.39±0.01	3449.55±596.93	0.13±0.00
98	*Chrysanthemi Indici Flos*	20	70	19,514.52±455.58	-15.15±7.34	0.51±0.03	0.38±0.01	3763.98±210.53	0.13±0.00
99	*Chrysanthemi Indici Flos*	20	80	16,586.02±529.24	-11.75±2.11	0.49±0.03	0.36±0.02	2879.53±323.88	0.11±0.00
100	*Chrysanthemi Indici Flos*	20	90	14,510.42±747.63	-17.23±1.11	0.65±0.12	0.31±0.02	2876.93±326.08	0.11±0.00
101	*Chrysanthemi Indici Flos*	30	50	22,782.45±655.56	-19.20±0.81	0.51±0.04	0.40±0.01	4603.39±456.17	0.13±0.00
102	*Chrysanthemi Indici Flos*	30	60	18,638.36±134.65	-12.52±1.04	0.46±0.03	0.38±0.00	3284.81±241.87	0.12±0.00
103	*Chrysanthemi Indici Flos*	30	70	15,576.19±423.12	-25.57±4.87	0.56±0.10	0.33±0.01	2889.39±440.96	0.11±0.00
104	*Chrysanthemi Indici Flos*	30	80	12,785.99±479.06	-48.95±2.51	0.85±0.09	0.27±0.01	2955.13±407.38	0.10±0.00
105	*Chrysanthemi Indici Flos*	40	30	16,872.44±1193.53	-200.40±20.87	0.96±0.03	0.28±0.01	4473.39±340.69	0.08±0.00
106	*Chrysanthemi Indici Flos**	40	35	16,869.92±1067.85	-160.43±26.77	1.59±1.03	0.31±0.00	8343.56±5799.84	0.09±0.00
107	*Chrysanthemi Indici Flos*	40	45	13,260.11±497.93	-45.78±18.42	0.90±0.13	0.29±0.00	3423.43±653.24	0.10±0.00
108	*Chrysanthemi Indici Flos*	40	40	15,573.77±452.27	-120.92±30.82	0.91±0.15	0.30±0.01	4232.00±709.89	0.09±0.00
109	*Magnoliae Flos*	20	55	21,429.42±657.29	-10.63±4.36	0.45±0.03	0.35±0.01	3386.95±394.02	0.13±0.01
110	*Magnoliae Flos*	20	65	20,940.75±850.21	-5.83±1.46	0.38±0.02	0.38±0.00	3024.98±72.11	0.13±0.00
111	*Magnoliae Flos*	20	75	20,010.96±811.18	-19.15±3.68	0.46±0.05	0.38±0.02	3422.81±162.07	0.13±0.00
112	*Magnoliae Flos*	20	85	16,263.37±612.50	-23.78±4.85	0.58±0.08	0.34±0.02	3183.49±318.79	0.11±0.00
113	*Magnoliae Flos*	30	60	18,936.84±1301.16	-8.71±0.87	0.40±0.03	0.37±0.00	2761.46±132.98	0.12±0.00
114	*Magnoliae Flos*	30	65	18,411.49±629.83	-15.83±0.63	0.45±0.05	0.36±0.01	2974.04±313.99	0.12±0.00
115	*Magnoliae Flos*	30	70	16,038.46±653.56	-16.73±3.93	0.44±0.01	0.34±0.01	2357.86±206.69	0.11±0.01
116	*Magnoliae Flos*	30	75	14,245.41±188.56	-24.29±3.66	0.52±0.08	0.30±0.00	2269.76±386.29	0.10±0.00
117	*Magnoliae Flos*	40	45	20,963.68±558.18	-25.08±5.72	0.44±0.07	0.36±0.01	3291.01±493.59	0.11±0.00
118	*Magnoliae Flos*	40	50	15,566.56±173.54	-25.86±0.96	0.45±0.02	0.32±0.01	2236.37±134.65	0.10±0.00
119	*Magnoliae Flos*	40	55	13,709.49±274.90	-35.87±1.97	0.88±0.11	0.29±0.01	3533.93±579.49	0.09±0.00
120	*Magnoliae Flos*	40	65	34,483.32±3484.81	-727.11±126.99	0.99±0.00	0.61±0.03	20849.17±3112.32	0.45±0.04
121	*Citri Reticulatae Pericarpium*	20	45	23,047.66±1741.80	-14.18±4.38	0.41±0.05	0.38±0.01	3564.73±617.63	0.12±0.00
122	*Citri Reticulatae Pericarpium*	20	55	20,998.51±510.14	-6.23±0.95	0.41±0.01	0.38±0.01	3252.13±171.37	0.12±0.00
123	*Citri Reticulatae Pericarpium*	20	65	16,110.94±1118.20	-15.63±2.19	0.42±0.02	0.36±0.02	2443.97±434.70	0.11±0.01
124	*Citri Reticulatae Pericarpium*	20	75	16,451.80±626.90	-15.11±0.95	0.50±0.09	0.36±0.02	2895.80±391.23	0.11±0.00
125	*Citri Reticulatae Pericarpium*	30	45	18,916.07±2158.67	-22.78±6.76	0.45±0.03	0.37±0.01	3194.54±502.23	0.12±0.01
126	*Citri Reticulatae Pericarpium*	30	55	25,133.94±338.13	-17.62±5.05	0.49±0.05	0.40±0.01	4811.31±312.50	0.13±0.00
127	*Citri Reticulatae Pericarpium*	30	65	13,477.46±590.04	-30.32±2.42	0.53±0.06	0.32±0.01	2228.30±257.69	0.10±0.00
128	*Citri Reticulatae Pericarpium*	30	75	10,959.43±300.65	-57.39±5.27	0.86±0.08	0.26±0.00	2422.43±291.23	0.09±0.00
129	*Citri Reticulatae Pericarpium**	40	25	14,195.79±1232.84	-683.75±131.60	0.65±0.36	0.24±0.01	2088.63±1011.68	0.06±0.00
130	*Citri Reticulatae Pericarpium**	40	35	12,702.00±588.10	-298.70±62.43	1.00±0.00	0.23±0.02	2954.68±136.99	0.07±0.00
131	*Citri Reticulatae Pericarpium*	40	45	11,799.35±1241.91	-322.74±165.53	1.00±0.00	0.24±0.01	2804.70±381.08	0.07±0.01
132	*Citri Reticulatae Pericarpium*	40	55	10,406.84±383.15	-241.11±9.25	0.99±0.00	0.23±0.00	2404.66±74.79	0.07±0.00
133	*Farfarae Flos*	20	55	20,269.90±9197.35	-6.56±90.36	0.47±0.26	0.37±0.01	3551.58±2243.55	0.13±0.01
134	*Farfarae Flos*	20	65	21,471.68±623.86	-15.60±17.19	0.38±0.08	0.37±0.02	2978.57±515.96	0.13±0.00
135	*Farfarae Flos*	20	75	19,250.69±424.52	-9.91±1.87	0.42±0.04	0.38±0.01	3073.62±306.54	0.12±0.00
136	*Farfarae Flos*	20	85	15,813.01±407.33	-8.07±0.24	0.43±0.04	0.37±0.02	2486.51±383.58	0.12±0.00
137	*Farfarae Flos*	30	35	22,861.22±2842.91	-20.51±6.01	0.39±0.06	0.37±0.02	3390.21±968.83	0.12±0.01
138	*Farfarae Flos*	30	45	19,501.94±1136.09	-11.50±3.89	0.45±0.04	0.38±0.00	3300.28±356.11	0.12±0.00

139	*Farfarae Flos*	30	55	16,834.48±771.41	-16.73±1.36	0.43±0.03	0.36±0.01	2626.12±223.60	0.11±0.00
140	*Farfarae Flos*	30	65	15,221.73±830.92	-14.63±2.84	0.52±0.05	0.36±0.01	2840.00±439.49	0.11±0.00
141	*Farfarae Flos*	40	35	19,284.21±1787.59	-44.09±9.46	0.46±0.04	0.36±0.01	3189.24±295.14	0.11±0.00
142	*Farfarae Flos*	40	40	15,404.83±1136.42	-31.42±2.18	0.65±0.09	0.33±0.02	3248.56±115.70	0.11±0.00
143	*Farfarae Flos*	40	45	14,758.46±1209.44	-39.04±4.69	0.57±0.07	0.33±0.01	2761.97±602.94	0.10±0.00
144	*Farfarae Flos*	40	55	12,622.30±740.44	-62.04±22.64	0.76±0.19	0.29±0.02	2711.11±352.39	0.10±0.01
145	*Bupleuri Radix*	20	45	21,431.38±308.84	-9.48±2.35	0.40±0.01	0.35±0.02	3052.08±230.56	0.12±0.00
146	*Bupleuri Radix*	20	55	22,125.73±678.50	-4.64±0.21	0.38±0.02	0.38±0.01	3198.81±171.16	0.13±0.00
147	*Bupleuri Radix*	20	65	19,846.12±823.59	-7.37±6.18	0.38±0.03	0.37±0.01	2762.03±312.64	0.12±0.00
148	*Bupleuri Radix*	20	75	18,222.88±1211.94	-14.70±7.58	0.40±0.05	0.37±0.01	2685.81±484.47	0.12±0.01
149	*Bupleuri Radix*	30	40	21,609.87±871.26	-14.51±12.59	0.43±0.07	0.37±0.01	3455.53±644.22	0.12±0.01
150	*Bupleuri Radix*	30	50	17,350.45±1311.36	-12.83±3.96	0.42±0.02	0.36±0.02	2615.09±313.30	0.11±0.00
151	*Bupleuri Radix*	30	60	15,194.23±623.70	-26.09±17.19	0.47±0.08	0.34±0.02	2423.22±515.78	0.11±0.00
152	*Bupleuri Radix*	30	70	12,814.37±246.58	-37.70±6.40	0.90±0.05	0.30±0.02	3451.18±117.41	0.10±0.00
153	*Bupleuri Radix*	40	30	17,034.40±656.47	-73.52±14.04	0.84±0.18	0.32±0.01	4583.11±1152.94	0.10±0.01
154	*Bupleuri Radix*	40	35	16,333.10±475.73	-86.20±16.15	0.66±0.09	0.33±0.01	3506.46±538.49	0.10±0.00
155	*Bupleuri Radix*	40	45	15,814.04±595.84	-34.36±6.18	0.49±0.10	0.33±0.01	2581.07±532.80	0.10±0.00
156	*Bupleuri Radix*	40	55	12,111.67±338.45	-74.84±15.80	0.94±0.09	0.26±0.01	2944.88±332.79	0.09±0.00
157	*Astragali Radix*	20	60	20,959.67±477.70	-9.07±4.98	0.37±0.03	0.37±0.01	2908.34±202.68	0.12±0.00
158	*Astragali Radix*	20	70	18,158.91±632.26	-9.70±1.82	0.43±0.04	0.37±0.01	2868.30±281.76	0.12±0.00
159	*Astragali Radix*	20	80	16,588.04±405.44	-13.14±7.46	0.54±0.12	0.37±0.05	3376.71±1200.37	0.12±0.01
160	*Astragali Radix*	20	90	15,019.80±1055.47	-23.67±1.27	0.52±0.08	0.35±0.03	2659.71±320.96	0.12±0.01
161	*Astragali Radix*	30	25	24,918.37±604.42	-18.41±7.41	0.42±0.01	0.34±0.01	3587.51±57.30	0.11±0.00
162	*Astragali Radix*	30	35	23,387.07±593.12	-11.16±3.34	0.47±0.12	0.40±0.04	4441.85±1679.89	0.13±0.02
163	*Astragali Radix*	30	45	20,659.64±1726.67	-12.66±5.35	0.43±0.04	0.38±0.01	3330.23±174.21	0.12±0.00
164	*Astragali Radix*	30	55	11,233.81±532.16	-82.33±8.14	0.98±0.03	0.26±0.01	2844.00±112.41	0.09±0.00
165	*Astragali Radix*	40	25	18,570.26±1073.95	-133.25±45.45	0.59±0.23	0.34±0.01	3641.34±1194.98	0.09±0.00
166	*Astragali Radix*	40	30	21,058.68±464.30	-48.55±37.54	0.43±0.00	0.36±0.01	3255.33±163.15	0.11±0.00
167	*Astragali Radix*	40	40	15,835.36±1354.39	-18.13±5.63	0.40±0.01	0.35±0.01	2212.13±281.62	0.11±0.00
168	*Astragali Radix*	40	45	12,189.59±1054.96	-50.18±11.31	0.71±0.08	0.30±0.01	2619.72±295.51	0.09±0.00
169	*Atractylodis Macrocephalae Rhizoma*	20	55	20,773.99±823.85	-4.55±0.40	0.39±0.02	0.38±0.01	3106.99±244.80	0.12±0.00
170	*Atractylodis Macrocephalae Rhizoma*	20	65	20,090.77±707.67	-8.74±10.00	0.43±0.32	0.37±0.02	2873.16±1427.91	0.13±0.01
171	*Atractylodis Macrocephalae Rhizoma*	20	75	16,597.43±442.59	-15.21±3.42	0.54±0.11	0.35±0.02	3110.75±445.33	0.11±0.00
172	*Atractylodis Macrocephalae Rhizoma*	20	85	13,345.28±539.99	-41.45±10.93	0.97±0.04	0.29±0.00	3800.17±232.53	0.10±0.00
173	*Atractylodis Macrocephalae Rhizoma*	30	55	19,750.09±462.49	-7.40±2.66	0.42±0.01	0.38±0.00	3082.27±96.66	0.12±0.00
174	*Atractylodis Macrocephalae Rhizoma*	30	65	13,899.26±950.22	-29.26±2.87	0.60±0.15	0.32±0.01	2659.91±613.78	0.10±0.00
175	*Atractylodis Macrocephalae Rhizoma*	30	75	11,015.31±289.45	-69.74±27.14	0.92±0.14	0.26±0.01	2626.57±480.13	0.10±0.00
176	*Atractylodis Macrocephalae Rhizoma*	30	85	13,169.53±648.20	-160.94±6.89	0.94±0.08	0.31±0.01	3815.58±487.05	0.13±0.01
177	*Atractylodis Macrocephalae Rhizoma*	40	35	15,999.05±1211.25	-27.33±7.58	0.49±0.05	0.35±0.01	2736.73±484.19	0.10±0.01
178	*Atractylodis Macrocephalae Rhizoma*	40	40	13,769.82±387.80	-30.64±6.90	0.58±0.05	0.32±0.01	2571.35±264.43	0.10±0.00
179	*Atractylodis Macrocephalae Rhizoma*	40	45	12,029.65±502.04	-47.77±1.45	0.85±0.07	0.28±0.01	2873.83±314.15	0.09±0.00
180	*Atractylodis Macrocephalae Rhizoma*	40	55	12,431.45±534.61	-163.87±18.31	0.99±0.00	0.27±0.01	3351.07±182.27	0.11±0.01
181	*Leonuri Herba*	20	60	23,632.33±1842.88	-5.61±3.22	0.47±0.05	0.41±0.01	4570.44±779.93	0.15±0.01
182	*Leonuri Herba*	20	70	18,830.80±214.36	-7.73±2.78	0.44±0.04	0.39±0.02	3206.87±427.57	0.12±0.00

183	*Leonuri Herba*	20	80	16,972.56±525.90	-20.41±5.18	0.42±0.01	0.36±0.01	2594.46±44.26	0.11±0.00
184	*Leonuri Herba*	20	90	13,950.18±325.86	-14.36±1.35	0.52±0.05	0.34±0.02	2477.86±180.78	0.11±0.00
185	*Leonuri Herba*	30	45	21,798.96±562.36	-15.81±6.33	0.47±0.04	0.40±0.01	4068.61±382.46	0.13±0.00
186	*Leonuri Herba*	30	55	18,448.29±417.00	-14.71±3.21	0.42±0.03	0.37±0.00	2821.29±125.95	0.12±0.00
187	*Leonuri Herba*	30	65	19,996.11±224.41	-10.87±8.23	0.44±0.05	0.39±0.00	3420.47±396.91	0.12±0.00
188	*Leonuri Herba*	30	75	12,757.80±682.50	-33.82±2.56	0.92±0.06	0.29±0.01	3445.69±313.93	0.10±0.00
189	*Leonuri Herba*	40	30	22,052.40±2011.41	-123.55±20.16	0.86±0.25	0.37±0.03	7155.96±2446.44	0.11±0.02
190	*Leonuri Herba*	40	40	18,584.34±880.42	-18.60±13.29	0.92±0.53	0.36±0.01	6014.42±3254.67	0.11±0.00
191	*Leonuri Herba*	40	50	12,857.76±513.59	-48.03±8.94	0.97±0.03	0.29±0.01	3654.61±287.57	0.09±0.00
192	*Leonuri Herba**	40	60	19,764.95±271.77	-196.56±7.93	0.99±0.00	0.38±0.02	7534.23±336.77	0.21±0.02
193	*Gardeniae Fructus*	20	50	23,254.59±424.24	-10.00±1.87	0.39±0.04	0.39±0.01	3516.76±306.52	0.13±0.00
194	*Gardeniae Fructus*	20	60	21,579.02±1756.25	-16.45±2.62	0.40±0.02	0.38±0.01	3223.12±141.17	0.12±0.00
195	*Gardeniae Fructus*	20	65	23,173.55±1207.59	-9.22±2.19	0.45±0.02	0.40±0.01	4118.55±198.55	0.13±0.00
196	*Gardeniae Fructus*	20	70	20,245.86±1352.77	-7.29±1.47	0.43±0.02	0.39±0.01	3363.47±387.26	0.12±0.00
197	*Gardeniae Fructus*	30	35	23,659.49±2061.34	-69.26±69.70	0.38±0.04	0.34±0.03	2983.48±425.80	0.12±0.01
198	*Gardeniae Fructus*	30	45	26,372.76±2025.47	-23.33±2.50	0.44±0.04	0.37±0.02	4232.62±97.67	0.13±0.01
199	*Gardeniae Fructus*	30	50	21,861.06±917.44	-45.48±7.72	0.50±0.04	0.36±0.01	3902.04±443.05	0.12±0.00
200	*Gardeniae Fructus*	30	55	19,033.00±686.14	-34.95±9.23	0.46±0.07	0.34±0.01	3034.27±564.64	0.11±0.00
201	*Gardeniae Fructus*	40	25	14,138.89±1225.23	-77.98±3.14	0.56±0.21	0.22±0.02	1670.68±332.88	0.07±0.00
202	*Gardeniae Fructus*	40	35	12,752.67±961.46	-184.39±0.32	0.89±0.12	0.21±0.01	2341.99±53.85	0.07±0.01
203	*Gardeniae Fructus*	40	45	10,861.13±298.47	-269.99±23.46	1.00±0.00	0.21±0.02	2322.05±242.52	0.06±0.01
204	*Gardeniae Fructus*	40	50	10,211.24±81.47	-212.70±39.94	1.00±0.00	0.20±0.01	1990.34±99.45	0.07±0.00
205	*Schisandrae Chinensis Fructus*	20	65	16,289.39±755.02	-13.59±2.11	0.41±0.01	0.37±0.01	2458.22±107.61	0.12±0.00
206	*Schisandrae Chinensis Fructus*	20	75	16,808.42±1186.66	-18.85±5.54	0.51±0.12	0.35±0.01	2949.83±656.74	0.12±0.00
207	*Schisandrae Chinensis Fructus*	20	80	13,754.34±326.24	-15.36±3.98	0.62±0.07	0.30±0.00	2503.80±214.94	0.11±0.00
208	*Schisandrae Chinensis Fructus*	20	85	13,417.33±588.13	-25.89±8.16	0.51±0.00	0.31±0.02	2631.89±136.93	0.10±0.00
209	*Schisandrae Chinensis Fructus*	30	45	21,237.65±563.80	-16.13±2.04	0.45±0.01	0.39±0.01	3744.67±256.73	0.12±0.00
210	*Schisandrae Chinensis Fructus*	30	55	16,908.64±158.89	-16.13±2.04	0.43±0.01	0.35±0.01	2533.34±73.24	0.11±0.00
211	*Schisandrae Chinensis Fructus*	30	60	15,305.19±536.65	-15.40±4.34	0.46±0.03	0.35±0.00	2464.07±144.34	0.11±0.00
212	*Schisandrae Chinensis Fructus*	30	65	12,392.61±152.90	-27.29±7.60	0.67±0.10	0.29±0.01	2446.23±403.76	0.10±0.00
213	*Schisandrae Chinensis Fructus*	40	30	19,989.35±316.94	-68.73±8.21	0.66±0.06	0.36±0.01	4779.65±372.86	0.11±0.00
214	*Schisandrae Chinensis Fructus*	40	35	18,459.74±1315.14	-69.45±13.29	0.48±0.04	0.35±0.00	3143.97±391.11	0.10±0.01
215	*Schisandrae Chinensis Fructus*	40	40	16,475.51±240.11	-51.80±15.51	0.56±0.08	0.35±0.01	3206.16±425.00	0.11±0.00
216	*Schisandrae Chinensis Fructus*	40	45	16,270.99±467.59	-20.47±5.12	0.48±0.05	0.35±0.01	2734.98±189.22	0.11±0.00
217	*Eucommiae Cortex*	20	70	20,961.26±484.00	-5.67±0.53	0.43±0.02	0.38±0.01	3428.05±315.26	0.13±0.00
218	*Eucommiae Cortex*	20	80	19,765.10±764.51	-8.22±0.83	0.42±0.04	0.39±0.00	3191.05±263.86	0.13±0.00
219	*Eucommiae Cortex*	20	85	16,975.40±680.98	-11.85±1.37	0.44±0.04	0.37±0.02	2762.68±426.55	0.12±0.01
220	*Eucommiae Cortex*	20	90	15,615.26±565.23	-20.47±2.12	0.52±0.03	0.35±0.00	2803.10±253.15	0.12±0.00
221	*Eucommiae Cortex*	30	30	24,983.56±885.97	-14.65±2.75	0.41±0.03	0.38±0.01	3874.06±381.53	0.12±0.00
222	*Eucommiae Cortex*	30	40	22,494.27±440.37	-8.12±2.76	0.41±0.02	0.40±0.01	3702.27±170.52	0.13±0.00
223	*Eucommiae Cortex*	30	50	20,488.53±1221.54	-9.71±1.55	0.45±0.05	0.41±0.01	3741.13±290.79	0.13±0.00
224	*Eucommiae Cortex*	30	60	16,086.20±707.14	-17.37±10.15	0.63±0.32	0.36±0.02	3511.74±1427.64	0.12±0.01
225	*Eucommiae Cortex*	40	30	20,760.91±1198.05	-367.32±28.88	0.90±0.12	0.27±0.01	5071.17±568.91	0.09±0.00
226	*Eucommiae Cortex*	40	35	20,691.64±498.80	-35.32±5.12	0.45±0.01	0.36±0.01	3373.72±32.66	0.11±0.00
227	*Eucommiae Cortex*	40	40	17,832.66±715.98	-66.86±24.29	0.78±0.24	0.35±0.01	4846.72±1383.43	0.11±0.00
228	*Eucommiae Cortex*	40	50	12,586.53±229.07	-47.82±3.95	0.75±0.21	0.31±0.01	2915.42±808.70	0.10±0.00
229	*Sophorae Flavescentis Radix*	20	55	23,914.38±314.42	-10.00±2.04	0.41±0.01	0.40±0.01	3906.86±122.19	0.13±0.00
230	*Sophorae Flavescentis Radix*	20	65	18,324.66±584.20	-13.04±4.26	0.41±0.03	0.37±0.01	2727.21±166.90	0.12±0.00
231	*Sophorae Flavescentis Radix*	20	75	14,881.99±92.07	-17.74±8.89	0.41±0.05	0.33±0.01	2021.19±272.07	0.10±0.00

232	*Sophorae Flavescentis Radix*	20	85	11,508.40±548.11	-44.11±11.77	0.89±0.08	0.27±0.01	2792.79±174.69	0.09±0.00
233	*Sophorae Flavescentis Radix*	30	35	21,693.56±4.35	-10.38±2.56	0.40±0.06	0.38±0.02	3273.71±613.55	0.12±0.00
234	*Sophorae Flavescentis Radix*	30	45	15,178.74±65.87	-17.80±6.38	0.42±0.03	0.34±0.00	2153.85±146.77	0.10±0.00
235	*Sophorae Flavescentis Radix*	30	55	14,724.15±103.84	-16.80±4.84	0.43±0.01	0.33±0.01	2081.23±145.87	0.10±0.00
236	*Sophorae Flavescentis Radix*	30	65	11,558.03±473.42	-87.38±33.30	0.94±0.11	0.27±0.01	2954.76±265.91	0.09±0.00
237	*Sophorae Flavescentis Radix**	40	20	18,188.65±1727.39	-418.05±55.77	1.00±0.01	0.25±0.02	4501.84±125.32	0.07±0.01
238	*Sophorae Flavescentis Radix*	40	30	15,321.60±165.03	-100.50±43.45	0.95±0.06	0.30±0.01	4419.26±215.74	0.09±0.00
239	*Sophorae Flavescentis Radix*	40	40	14,235.07±265.17	-86.60±29.39	0.95±0.04	0.32±0.02	4315.23±267.94	0.10±0.02
240	*Sophorae Flavescentis Radix*	40	50	12,290.94±498.07	-76.74±12.59	1.00±0.00	0.28±0.00	3453.99±131.55	0.09±0.01
241	*Gastrodiae Rhizoma*	20	60	19,900.58±1301.12	-26.49±0.87	0.70±0.03	0.37±0.00	5133.05±132.26	0.12±0.00
242	*Gastrodiae Rhizoma*	20	70	18,119.84±751.92	-12.77±2.23	0.40±0.06	0.36±0.01	2647.29±434.77	0.12±0.00
243	*Gastrodiae Rhizoma*	20	80	19,900.31±1301.04	-26.49±1.00	0.70±0.03	0.37±0.00	5133.60±132.51	0.12±0.00
244	*Gastrodiae Rhizoma*	20	90	14,060.56±596.48	-31.21±4.66	0.67±0.14	0.31±0.01	2865.19±493.08	0.11±0.00
245	*Gastrodiae Rhizoma*	30	40	22,091.91±687.69	-11.32±2.97	0.38±0.04	0.37±0.01	3086.07±336.20	0.11±0.00
246	*Gastrodiae Rhizoma*	30	50	19,105.27±787.38	-23.31±3.31	0.53±0.04	0.36±0.00	3622.03±136.95	0.11±0.00
247	*Gastrodiae Rhizoma*	30	60	16,141.85±626.42	-23.61±1.51	0.46±0.05	0.35±0.01	2604.52±379.40	0.11±0.00
248	*Gastrodiae Rhizoma*	30	70	13,071.42±552.89	-42.42±9.27	0.92±0.07	0.29±0.00	3518.27±383.98	0.10±0.00
249	*Gastrodiae Rhizoma*	40	25	20,874.44±799.88	-213.35±14.59	0.97±0.05	0.29±0.01	5834.09±310.67	0.09±0.00
250	*Gastrodiae Rhizoma*	40	35	18,613.53±1112.52	-109.40±9.67	0.72±0.24	0.32±0.00	4298.29±1694.72	0.09±0.00
251	*Gastrodiae Rhizoma*	40	45	13,809.80±267.39	-68.48±16.44	0.81±0.20	0.28±0.01	3150.42±700.57	0.09±0.00
252	*Gastrodiae Rhizoma*	40	55	12,283.56±163.15	-135.44±34.63	0.96±0.06	0.26±0.01	3091.70±131.84	0.09±0.00
253	*Artemisiae Argyi Folium*	20	55	24,057.43±2974.83	-5.36±1.21	0.44±0.04	0.37±0.01	4003.04±727.88	0.14±0.01
254	*Artemisiae Argyi Folium*	20	65	19,746.29±793.91	-6.06±0.73	0.44±0.05	0.36±0.01	3127.81±313.40	0.13±0.00
255	*Artemisiae Argyi Folium*	20	75	18,584.00±2108.03	-6.45±0.26	0.42±0.01	0.37±0.00	2880.76±371.32	0.13±0.01
256	*Artemisiae Argyi Folium*	20	85	17,382.25±1380.81	-8.11±-7.97	0.47±0.03	0.38±0.01	3053.53±520.21	0.13±0.01
257	*Artemisiae Argyi Folium*	30	40	21,812.88±2771.45	-11.60±15.60	0.50±0.08	0.39±0.01	4250.08±974.05	0.13±0.01
258	*Artemisiae Argyi Folium*	30	50	18,919.86±640.69	-8.54±4.34	0.42±0.01	0.37±0.01	2935.95±124.95	0.12±0.00
259	*Artemisiae Argyi Folium*	30	60	17,287.06±603.31	-9.36±2.40	0.46±0.01	0.38±0.01	3003.85±101.35	0.12±0.00
260	*Artemisiae Argyi Folium*	30	70	13,841.81±466.97	-20.04±2.40	0.61±0.07	0.32±0.00	2705.21±222.02	0.11±0.00
261	*Artemisiae Argyi Folium*	40	25	19,160.30±1447.91	-116.63±24.08	0.80±0.21	0.33±0.01	5171.71±1581.25	0.10±0.01
262	*Artemisiae Argyi Folium*	40	35	17,720.40±871.50	-34.59±12.59	0.52±0.07	0.36±0.01	3338.82±644.33	0.11±0.01
263	*Artemisiae Argyi Folium*	40	45	12,804.41±290.47	-37.41±5.59	0.89±0.05	0.30±0.01	3419.98±273.04	0.10±0.00
264	*Artemisiae Argyi Folium*	40	55	12,587.07±1289.74	-32.09±4.32	0.67±0.09	0.30±0.02	2503.34±215.00	0.10±0.01
265	*Siegesbeckiae Herba*	20	65	15,980.40±1193.62	-25.56±20.87	0.46±0.03	0.35±0.01	2540.49±340.02	0.11±0.00
266	*Siegesbeckiae Herba*	20	75	19,416.86±810.02	-5.87±0.59	0.42±0.03	0.38±0.00	3099.84±95.18	0.12±0.00
267	*Siegesbeckiae Herba*	20	85	15,044.44±538.90	-9.41±1.94	0.45±0.05	0.32±0.01	2186.49±237.39	0.11±0.00
268	*Siegesbeckiae Herba*	20	95	13,414.19±229.20	-16.25±5.43	0.68±0.24	0.29±0.02	2650.91±769.99	0.11±0.00
269	*Siegesbeckiae Herba*	30	40	24,431.29±2185.73	-5.06±1.68	0.43±0.04	0.39±0.01	4079.77±96.10	0.12±0.01
270	*Siegesbeckiae Herba*	30	50	21,502.50±547.38	-7.91±2.32	0.45±0.01	0.39±0.02	3746.89±323.96	0.12±0.01
271	*Siegesbeckiae Herba*	30	60	17,004.52±358.74	-9.60±1.23	0.44±0.03	0.36±0.01	2686.30±250.92	0.11±0.01
272	*Siegesbeckiae Herba*	30	70	13,041.48±696.98	-22.37±10.15	0.70±0.21	0.29±0.01	2635.89±644.80	0.10±0.00
273	*Siegesbeckiae Herba*	40	25	25,138.37±3385.29	-64.19±25.27	0.45±0.03	0.35±0.01	3979.64±979.13	0.11±0.01
274	*Siegesbeckiae Herba*	40	35	22,040.52±905.91	-32.61±6.08	0.46±0.02	0.38±0.01	3829.11±196.33	0.11±0.00
275	*Siegesbeckiae Herba*	40	45	17,728.64±423.63	-24.42±3.65	0.49±0.03	0.38±0.01	3257.15±274.98	0.11±0.00
276	*Siegesbeckiae Herba**	40	55	26,196.16±955.14	-152.15±28.98	0.97±0.04	0.52±0.02	13182.70±1539.49	0.35±0.03
277	*Rhei Radix Et Rhizoma*	20	65	21,382.85±1502.39	-10.10±1.71	0.40±0.02	0.37±0.00	3200.51±88.08	0.12±0.00
278	*Rhei Radix Et Rhizoma*	20	75	16,497.74±505.47	-16.60±1.76	0.41±0.06	0.33±0.00	2233.90±309.18	0.10±0.00
279	*Rhei Radix Et Rhizoma*	20	85	12,684.03±38.73	-28.69±4.90	0.85±0.12	0.28±0.01	2996.16±395.54	0.09±0.00
280	*Rhei Radix Et Rhizoma*	20	95	11,692.18±135.21	-39.93±5.22	0.89±0.07	0.25±0.01	2638.29±223.61	0.09±0.00

281	*Rhei Radix Et Rhizoma*	30	40	24,672.24±2668.14	-23.84±6.51	0.39±0.02	0.37±0.02	3529.07±730.01	0.12±0.01
282	*Rhei Radix Et Rhizoma*	30	50	19,989.19±452.76	-27.95±30.82	0.54±0.15	0.37±0.01	3425.62±709.10	0.11±0.00
283	*Rhei Radix Et Rhizoma*	30	60	13,735.18±63.84	-29.88±8.08	0.79±0.19	0.29±0.01	3110.33±630.47	0.09±0.00
284	*Rhei Radix Et Rhizoma*	30	70	12,982.91±310.77	-58.29±17.69	0.99±0.02	0.28±0.01	3631.86±149.34	0.09±0.00
285	*Rhei Radix Et Rhizoma*	40	30	21,715.95±1642.08	-164.00±22.96	0.91±0.14	0.33±0.02	6529.12±1335.89	0.10±0.01
286	*Rhei Radix Et Rhizoma*	40	40	16,790.39±1470.40	-120.13±4.76	1.00±0.00	0.32±0.05	5333.13±1313.16	0.09±0.02
287	*Rhei Radix Et Rhizoma*	40	50	16,619.63±535.20	-87.13±11.26	0.96±0.01	0.32±0.01	5046.44±367.67	0.09±0.00
288	*Rhei Radix Et Rhizoma*	40	60	11,311.82±673.05	-228.87±25.47	0.99±0.00	0.24±0.01	2726.97±298.32	0.09±0.01
289	*Eriobotryae Folium*	20	50	24,778.16±1646.53	-4.56±2.21	0.49±0.18	0.42±0.06	5289.35±2640.19	0.15±0.05
290	*Eriobotryae Folium*	20	60	20,676.65±702.73	-18.01±5.90	0.37±0.02	0.34±0.00	2616.67±229.28	0.10±0.00
291	*Eriobotryae Folium*	20	70	16,629.87±315.74	-13.97±5.64	0.51±0.14	0.37±0.05	3167.37±1291.79	0.13±0.05
292	*Eriobotryae Folium*	20	80	12,120.67±93.57	-24.69±2.19	0.58±0.09	0.28±0.01	1983.67±214.19	0.09±0.00
293	*Eriobotryae Folium*	30	40	23,364.60±1088.19	-30.89±4.11	0.40±0.02	0.36±0.01	3348.58±295.74	0.11±0.00
294	*Eriobotryae Folium*	30	50	19,706.87±451.85	-22.03±5.14	0.45±0.05	0.36±0.01	3148.71±363.51	0.11±0.00
295	*Eriobotryae Folium*	30	60	13,147.21±685.33	-37.64±5.81	0.61±0.17	0.30±0.01	2412.96±760.58	0.09±0.00
296	*Eriobotryae Folium*	30	70	13,155.02±325.68	-125.00±25.35	0.99±0.01	0.28±0.01	3603.71±56.14	0.11±0.00
297	*Eriobotryae Folium*	40	30	19,526.40±1917.72	-170.77±34.88	0.83±0.26	0.27±0.00	4279.80±992.42	0.08±0.00
298	*Eriobotryae Folium*	40	40	12,395.56±464.58	-203.31±37.54	1.00±0.00	0.24±0.01	2907.15±163.14	0.07±0.00
299	*Eriobotryae Folium*	40	45	13,854.43±274.67	-145.39±1.49	1.00±0.00	0.26±0.01	3564.02±24.24	0.07±0.00
300	*Eriobotryae Folium*	40	50	10,605.10±628.05	-203.45±29.11	1.00±0.00	0.22±0.00	2341.74±172.33	0.07±0.00

Notes—R: Surface roughness of the extrudates. E1: Extrudate cannot be extruded smoothly and the total amount of extrudate obtained is less than half of the amount fed. E2: Extrudate can be extruded smoothly with smooth surface. E3: Extrudate can be extruded smoothly with rough surface. E4: Extrudate can be extruded smoothly. But it's clumped together. S1: The extrudate after rounding becomes fine powder. S2: The extrudate is rounded to obtain pellets in the form of rods, dumbbells or double balls. S3: The extrudate is rounded to obtain pellets in the form of spherical. S4: The extrudate after rounding becomes the large ball or irregularly shaped mass.
